# Corrigendum to: Propofol differentially induces unconsciousness and respiratory depression through distinct interactions between GABAA receptor and GABAergic neuron in corresponding nuclei

**DOI:** 10.3724/abbs.2024135

**Published:** 2023-08-07

**Authors:** Junli Jiang, Yingfu Jiao, Po Gao, Wen Yin, Wei Zhou, Yunchun Zhang, Yanjun Liu, Daxiang Wen, Yuan Wang, Liang Zhou, Tian Yu, Weifeng Yu

Acta Biochim Biophys Sin 2021, 53(8):1076–1087.

https://doi.org/10.1093/abbs/gmab084

In the version of this article initially published, an error was found in the calculation of propofol’s concentrations in electrophysiological recordings. The correct propofol concentrations used in this article is 5 μM and 10 μM. The authors apologize for this error and any confusion it may have caused.

